# Synthesis of *C*_3_-symmetric star-shaped molecules containing α-amino acids and dipeptides via Negishi coupling as a key step

**DOI:** 10.3762/bjoc.15.33

**Published:** 2019-02-08

**Authors:** Sambasivarao Kotha, Saidulu Todeti

**Affiliations:** 1 Department of Chemistry, Indian Institute of Technology-Bombay, Powai, Mumbai-400076, India, Fax: +91(22)-2572 7152

**Keywords:** amino acids, cyclotrimerization, Negishi coupling, peptide

## Abstract

We demonstrate a new synthetic strategy toward star-shaped *C*_3_-symmetric molecules containing α-amino acid (AAA) derivatives and dipeptides. In this regard, trimerization and Negishi cross-coupling reactions are used as the key steps starting from readily available 4’-iodoacetophenone and L-serine. These *C*_3_-symmetric molecules containing AAA moieties are useful to design new ligands suitable for asymmetric synthesis and peptide dendrimers.

## Introduction

Optically active *C*_3_-symmetric molecules are valuable synthons to design dendrimers, chiral ligands, polymers, and supramolecules [1–4]. In this regard, 1,3,5-triarylbenzene derivatives are helpful to design star-shaped α-amino acids (AAAs) and they play an important role in biological systems. The tumor necrosis factor (TNF) superfamily belongs to trimeric ligands that form in the shape of *C*_3_-symmetric molecules [5]. Trimeric proteins containing star-shaped compounds are also involved in the complex interactions between cells and pathogens, e.g., the human immunodeficiency virus (HIV-1) [6]. The HIV-1 envelope protein is present as a *C*_3_-symmetric trimer on the viruses’ surface [7], and the virus entry into the cell is mediated by its interactions with cellular receptors. To explore the structural and chemical nature of protein–protein interactions, synthetic peptides and unnatural AAAs [8–15] can be useful as molecular tools. Moreover, *C*_3_-symmetric peptides are valuable in studying the molecular interactions involving proteins that are derived from trimers and synthetic access to such amino acids is vital. In this regard, new star-shaped *C*_3_-symmetric molecules [16–26] have been used in photovoltaics [27–28], organic light-emitting diodes (OLEDs) [29–30], organic field-effect transistors (OFETs) [31–32] and electroluminescent devices [33]. To address these challenges, we [34] and others [35–36] have synthesized functionalized *C*_3_-symmetric molecules containing amino acids and peptides.

**Figure 1:**
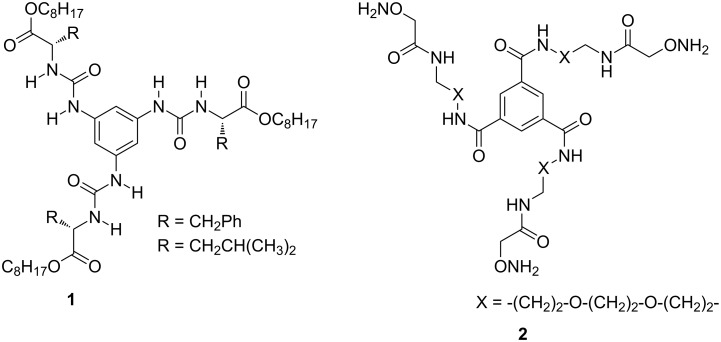
Exemplar *C*_3_-symmetric peptide scaffolds reported in the literature.

The Negishi cross coupling [37–38] is a reliable synthetic method, which involves palladium or nickel-catalyzed coupling of organozinc reagents [39–40] with various halo derivatives (e.g., aryl, vinyl, benzyl, or allyl) and has a broad scope to assemble diverse targets. This reaction was first reported in 1977, and it is an elegant and versatile method that allows the preparation of biaryls and olefins in good yields. To the best of our knowledge only a limited number of reports is available for the synthesis of *C*_3_-symmetric peptides (Figure 1) [8,41]. To fill this gap, we have explored a new synthetic strategy to star-shaped *C*_3_-symmetric AAA derivatives and peptides by using trimerization and the Negishi cross coupling as key steps.

## Results and Discussion

**Scheme 1:**
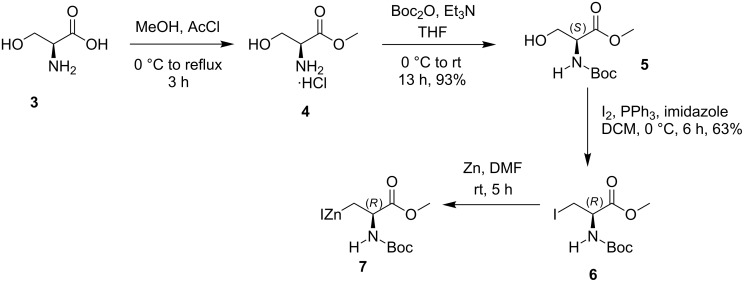
Preparation of compound **7** from L-serine (**3**).

The required zinc insertion compound **7** was prepared from L-serine (**3**). Thus, commercially available L-serine (**3**) was treated with acetyl chloride in methanol to give methyl ester **4**, which was subjected to *N*-Boc protection with di-*tert*-butyl dicarbonate (Boc_2_O) and triethylamine in tetrahydrofuran (THF) to obtain the *N*-Boc-serine methyl ester (**5**) in 93% yield [42]. Afterwards, the protected methyl ester **5** was subjected to iodination in the presence of iodine (I_2_), triphenylphosphane (PPh_3_) and imidazole in CH_2_Cl_2_ at 0 °C to deliver the iodo derivative **6** in 63% yield [43–44]. Finally, the iodo compound **6** was treated with freshly activated Zn in DMF at room temperature to afford the zinc insertion product **7** (Scheme 1) [43].

**Scheme 2:**
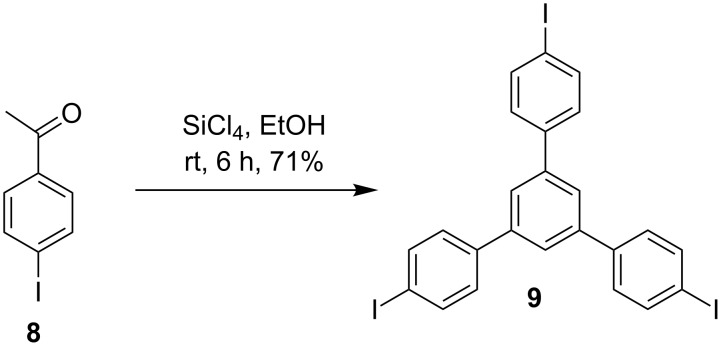
Preparation of the trimerized product **9**.

With the organozinc compound 7 at hand we turned to the synthesis of the halide component for the attempted Negishi coupling. For this 4-iodoacetophenone (**8**) was treated with silicon tetrachloride and ethanol (SiCl_4_/EtOH) at room temperature for 6 h to produce the iodonated trimerized product **9** in 71% yield (Scheme 2) [45–46].

**Scheme 3:**
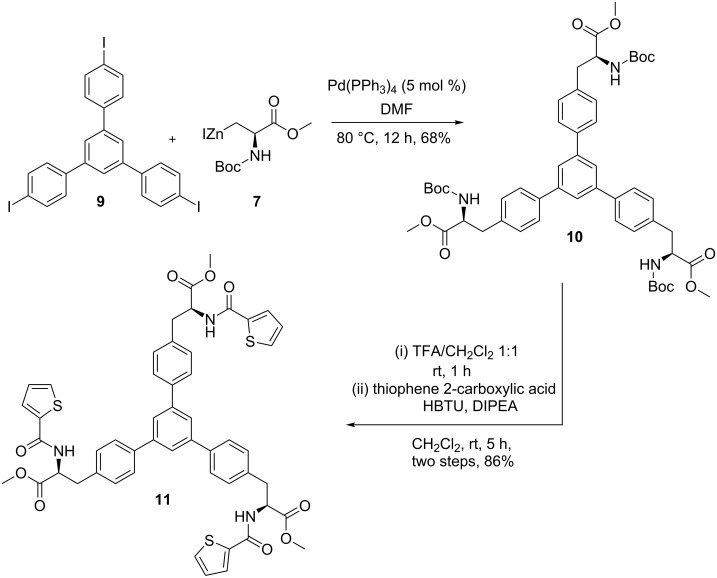
Synthesis of compound **11** via Negishi cross-coupling reaction.

Then, the organozinc reagent **7** was coupled with triiodo derivative **9** in the presence of tetrakis(triphenylphosphane)palladium(0) (Pd(PPh_3_)_4_) as catalyst to provide the Negishi coupling product **10** (68%). Having the trimeric AAA derivative **10** in hand, it was treated with trifluoroacetic acid (TFA) in CH_2_Cl_2_ (1:1) at room temperature for 1 h to deliver the Boc-deprotected compound. Then, without further purification the deprotected product was directly treated with thiophene-2-carboxylic acid in the presence of 2-(1*H*-benzotriazol-1-yl)-1,1,3,3-tetramethyluronium hexaﬂuorophosphate (HBTU) and *N*,*N*-diisopropylethylamine (DIPEA) in CH_2_Cl_2_ at room temperature for 5 h to give trimer **11** in 86% yield (Scheme 3).

**Scheme 4:**
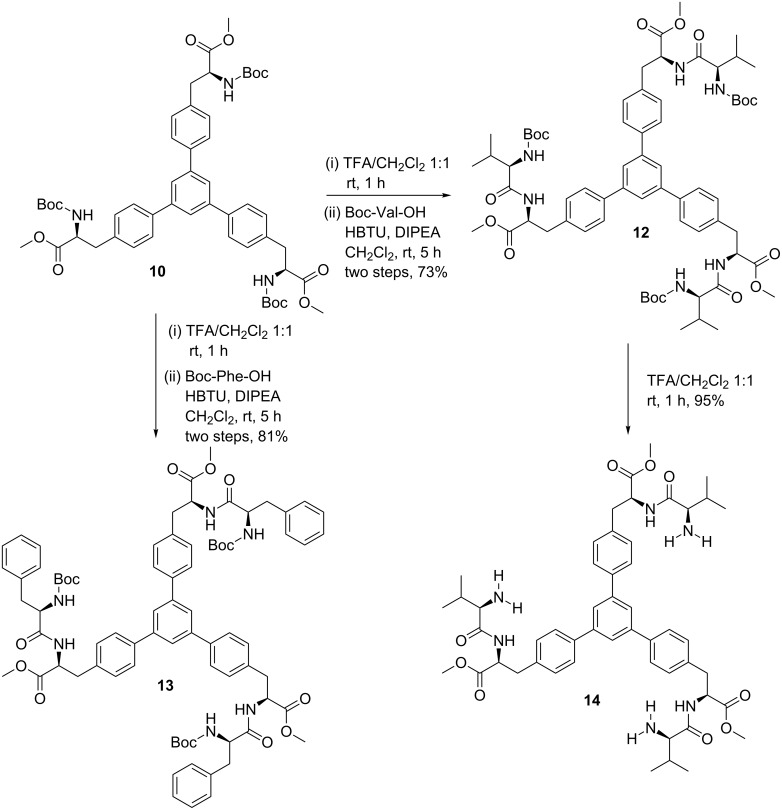
Synthesis of *C*_3_-symmetric trimers **12**, **13** and **14**.

In addition, different amino acids were incorporated in the star-shaped molecule. In this regard, the Negishi cross-coupling product **10** was treated with TFA in CH_2_Cl_2_ at room temperature for 1 h to give the Boc-deprotection product, which was directly treated with Boc-Val-OH or Boc-Phe-OH in the presence of HBTU and DIPEA in CH_2_Cl_2_ at room temperature for 5 h to give trimeric derivatives **12** (73%) and **13** (81%), respectively. Further, trimer **12** was subjected to another Boc-deprotection to give the tris-amine **14** in 95% yield (Scheme 4).

## Conclusion

We have demonstrated a simple synthetic strategy toward star-shaped molecules containing unusual AAA units through cyclotrimerization and Negishi cross-coupling reaction as key steps under operationally simple reaction conditions. Here, we have used the readily available starting materials 4-iodoacetophenone (**8**) and L-serine (**3**). The *C*_3_-symmetric building blocks prepared were coupled with different AAAs to produce the *C*_3_-symmetric dipeptide trimers.

## Experimental

### General procedure

Commercially available starting materials were used without further purification. Analytical thin layer chromatography (TLC) was performed on 7.5 × 2.5 cm glass plates coated with Acme’s silica gel GF254 (containing 13% calcium sulfate as binder) by using a suitable mixture of ethyl acetate and petroleum ether for development. The Negishi coupling was performed in oven-dried glassware under argon or nitrogen atmosphere and the transfer of moisture-sensitive materials was carried out in a glovebox by using standard syringe–septum techniques. All purchased solvents (CH_2_Cl_2_, THF, acetonitrile, and DMF) were dried over calcium hydride (CaH_2_) or sodium. Column chromatography was performed by using Acme’s silica gel (100–200 mesh) with an appropriate mixture of ethyl acetate, petroleum ether methanol and dichloromethane. The coupling constants (*J*) are given in hertz (Hz) and chemical shifts are denoted in parts per million (ppm) downfield from internal standard, tetramethylsilane (TMS). The abbreviations, s, d, t, q, m, and dd refer to singlet, doublet, triplet, quartet, multiplet, and doublet of doublets, respectively. Infrared (IR) spectra were recorded on a Nicolet Impact-400 FTIR spectrometer. Specific rotation experiments were measured at 589 nm (Na) and 25 °C (HPLC, CHCl_3_ stabilized with 0.7–1.0% ethanol). Proton nuclear magnetic resonance (^1^H NMR, 400 MHz and 500 MHz) spectra and carbon nuclear magnetic resonance (^13^C NMR, 100 MHz and 125 MHz) spectra were recorded on a Bruker spectrometer. The high-resolution mass measurements were carried out by using electrospray ionization (ESI) spectrometer. Melting points were recorded on a Veego melting point apparatus.

#### Negishi coupling product **10**

Zinc (Zn) dust was activated by using 3 M aq HCl, then filtered and washed with water (until neutral pH) followed by acetone. Large particles were crushed until a fine powder was formed and transferred into a round-bottomed flask and dried under vacuum with heating and the flask was filled with nitrogen. A portion of the activated Zn dust (500 mg, 7.65 mmol, 3 equiv) was cooled to room temperature. Then, iodo compound **6** (842 mg, 2.55 mmol) was dissolved in DMF (10 mL) and added dropwise to the freshly activated Zn powder under a nitrogen atmosphere and the suspension was stirred at room temperature for 3 h. After completion of Zn insertion reaction, stirring was stopped and the solid was allowed to settle down. The supernatant was carefully transferred to a suspension of triiodo derivative **9** (500 mg, 0.73 mmol) in DMF (10 mL) at room temperature. Five mol % tetrakis(triphenylphosphane)palladium (Pd(PPh_3_)_4_) was added to this mixture under inert atmosphere and the reaction mixture stirred at 80 °C for 12 h. The reaction mixture was cooled to room temperature and washed with water, brine (3 × 15 mL), 1 M aq Na_2_S_2_O_3_ solution and extracted with EtOAc (3 × 10 mL). The combined organic layers were dried over Na_2_SO_4_ and concentrated at reduced pressure. The crude was purified by silica gel column chromatography (30% ethyl acetate/petroleum ether) to afford the Negishi coupling product **10** (458 mg, 68%) as a colorless solid. *R*_f_ = 0.73 (3:7 ethyl acetate/petroleum ether), [α]_D_^25^ +7.78 (*c* 1.0, CHCl_3_); ^1^H NMR (400 MHz, CDCl_3_) δ 7.75 (s, 3H), 7.64 (d, *J* = 8.0 Hz, 6H), 7.28 (d, *J* = 8.0 Hz, 6H), 5.14 (d, *J* = 8.0 Hz, 3H), 4.67 (d, *J* = 6.8 Hz, 3H), 3.77 (s, 9H), 3.24–3.11 (m, 6H), 1.45 (s, 27H) ppm; ^13^C NMR (100 MHz, CDCl_3_) δ 172.4, 155.2, 141.9, 139.8, 135.5, 129.9, 127.4, 124.9, 80.0, 54.5, 52.3, 38.0, 28.3 ppm; HRMS–ESI (Q-Tof, *m*/*z*): [M + Na]^+^ calcd for C_51_H_63_N_3_NaO_12_, 932.4304; found, 932.4302; IR (neat) 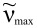: 3661, 2349, 1716, 1495, 1163, 1044, 755 cm^−1^.

#### General procedure for the mono- and dipeptide products **11**, **12** and **13**

Negishi coupling product **10** was dissolved in dichloromethane/trifluoroacetic acid (CH_2_Cl_2_/TFA 1:1) and the reaction mixture was stirred at room temperature for 1 h. Then, the mixture was concentrated at reduced pressure to remove the solvent and dried under vacuum. Later, without further purification the Negishi coupling deprotection product was reacted with 3 equiv of thiophene 2-carboxylic acid or amino acids (*N*-Boc-L-valine or Boc-Phe-OH) in the presence of *N*,*N*-diisopropylethylamine (DIPEA, 4 equiv), 2-(1*H*-benzotriazol-1-yl)-1,1,3,3-tetramethyluronium hexaﬂuorophosphate (HBTU, 9 equiv) in CH_2_Cl_2_. Afterwards, the reaction mixture was stirred at room temperature for 5 h under an inert atmosphere. After completion of the reaction, the mixture was washed with water, brine (3 × 10 mL) and extracted with CH_2_Cl_2_ (2 × 10 mL). The combined organic layer was dried over Na_2_SO_4_ and concentrated at reduced pressure. The crude product was purified by silica gel column chromatography (80% ethyl acetate/petroleum ether) to afford the *C*_3_-symmetric mono- and dipeptide derivatives **11**, **12** and **13**, respectively.

#### Peptide derivative **11**

Colorless solid; yield 86% (89 mg, starting from 100 mg of **10**); *R*_f_ = 0.46 (7:3 ethyl acetate/petroleum ether); mp 156–158 °C; [α]_D_^25^ +25.07 (*c* 1.0, CHCl_3_); ^1^H NMR (500 MHz, CDCl_3_) δ 7.71 (s, 3H), 7.60 (d, *J* = 8.0 Hz, 6H), 7.48 (q, *J* = 3.5 Hz, 6H), 7.24 (d, *J* = 8.0 Hz, 6H), 7.04 (t, *J* = 4.5 Hz, 3H), 6.64 (d, *J* = 7.5 Hz, 3H), 5.10 (q, *J* = 5.5 Hz, 3H), 3.79 (s, 9H), 3.35–3.24 (m, 6H) ppm; ^13^C NMR (125 MHz, CDCl_3_) δ 172.0, 161.5, 141.9, 139.9, 138.2, 135.3, 130.6, 129.9, 128.7, 127.8, 127.6, 125.0, 53.6, 52.6, 37.7 ppm; HRMS–ESI (Q-Tof, *m*/*z*): [M + H]^+^ calcd for C_51_H_46_N_3_O_9_S_3_, 940.2391; found, 940.2392; IR (neat) 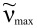: 3769, 3327, 2932, 1664, 1169, 759 cm^−1^.

#### Dipeptide **12**

Colorless solid; yield 73% (97 mg, starting from 100 mg of **10**); *R*_f_ = 0.59 (6:4 ethyl acetate/petroleum ether); mp <230 °C (dec); [α]_D_^25^ +20.58 (*c* 1.0, CHCl_3_); ^1^H NMR (400 MHz, CDCl_3_) δ 7.69 (s, 3H), 7.59 (d, *J* = 8.0 Hz, 6H), 7.21 (d, *J* = 7.6 Hz, 6H), 6.56 (d, *J* = 6.8 Hz, 3H), 5.12 (d, *J* = 7.2 Hz, 3H), 4.91 (d, *J* = 6.4 Hz, 3H), 3.95 (s, 3H), 3.72 (s, 9H), 3.16 (s, 6H), 2.09 (d, *J* = 6.0 Hz, 3H), 1.41 (s, 27 H), 0.92 (d, *J* = 6.8 Hz, 9H), 0.87 (d, *J* = 4.0 Hz, 9H) ppm; ^13^C NMR (100 MHz, CDCl_3_) δ 171.8, 171.5, 155.9, 141.9, 139.9, 135.3, 129.8, 127.5, 124.9, 79.9, 60.0, 53.2, 52.4, 37.8, 31.0, 28.4, 19.3, 17.8 ppm; HRMS–ESI (Q-Tof, *m*/z): [M + Na]^+^ calcd for C_66_H_90_N_6_NaO_15_, 1229.6356; found, 1229.6359; IR (neat) 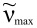: 3342, 2938, 2332, 1742, 1635, 1534, 1213, 754 cm^−1^.

#### Dipeptide **13**

Colorless solid; yield 81% (96 mg, starting from 80 mg of **10**); *R*_f_ = 0.73 (6:4 ethyl acetate/petroleum ether); mp 204–206 °C; ^1^H NMR (500 MHz, CDCl_3_) δ 7.70 (s, 3H), 7.56 (d, *J* = 8.0 Hz, 6H), 7.27 (d, *J* = 7.6 Hz, 6H), 7.20 (t, *J* = 5.6 Hz, 9H), 7.10 (d, *J* = 8.0 Hz, 6H), 6.35 (d, *J* = 6.80 Hz, 3H), 4.98 (br, 3H), 4.83 (d, *J* = 6.0 Hz, 3H), 4.35 (d, *J* = 5.20 Hz, 3H), 3.70 (s, 9H), 3.10–3.00 (m, 12H), 1.34 (s, 27H) ppm; ^13^C NMR (100 MHz, CDCl_3_) δ 171.5, 171.0, 155.4, 142.0, 140.0, 136.6, 135.2, 129.9, 129.5, 128.8, 127.6, 127.1, 125.0, 80.4, 55.9, 53.5, 52.5, 38.5, 37.8, 28.4 ppm; HRMS–ESI (Q-Tof, *m/z*): [M + Na]^+^ calcd for C_78_H_90_N_6_NaO_15_, 1373.6356; found, 1373.6359; IR (neat) 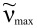: 3738, 3644, 2919, 2850, 2343, 1666, 1517, 814, 751 cm^−1^.

#### Trisamine derivative **14**

Compound **12** (95 mg, 0.07 mmol) was dissolved in CH_2_Cl_2_/TFA 1:1 and this mixture was stirred at room temperature for 1 h. At the conclusion of the reaction (TLC monitoring), the reaction mixture was concentrated at reduced pressure and dried under vacuum. The crude product was purified by silica gel column chromatography (2% MeOH/CHCl_3_) to obtain the Boc-deprotection product **14** (68 mg, 95%) as a colorless solid. *R*_f_ = 0.46 (0.5:9.5 methanol/chloroform); mp: **<**250 °C (dec); [α]_D_^24^ +0.97 (*c* 1.0, CHCl_3_); ^1^H NMR (500 MHz, CDCl_3_) δ 7.75 (s, 3H), 7.67 (d, *J* = 7.5 Hz, 6H), 7.39 (d, *J* = 8.0 Hz, 6H), 3.75 (d, *J* = 4.5 Hz, 3H), 3.71 (s, 9H), 3.28–3.24 (m, 3H), 3.13–3.09 (m, 3H), 2.25 (q, *J* = 6.0 Hz, 3H), 1.27 (s, 3H), 1.08 (d, *J* = 6.5 Hz, 9H), 1.04 (d, *J* = 7.0 Hz, 9H) ppm; ^13^C NMR (125 MHz, CDCl_3_) δ 173.5,170.4, 143.9, 141.5, 138.1, 131.4, 129.0, 126.1, 60.0, 56.1, 38.4, 32.2, 19.4, 18.1 ppm; HRMS–ESI (Q-Tof *m/z*): [M + H]^+^ calcd for C_51_H_67_N_6_O_9_, 907.4964; found, 907.4963; IR (neat) 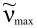: 3779, 3240, 2928, 1606, 1596, 1434, 783, 505 cm^−1^.

## Supporting Information

File 1 Copies of ^1^H, ^13^C NMR and HRMS spectra of new compounds.
