# Supplementary material for: Synthesis of C3-symmetric star-shaped molecules containing α-amino acids and dipeptides via Negishi coupling as a key step
Source: Beilstein J Org Chem. 2019 Feb 8;15:371–7. doi: 10.3762/bjoc.15.33 (PMC6369998; doi:10.3762/bjoc.15.33)

BEILSTEIN JOURNAL OF ORGANIC CHEMISTRY

Supporting Information

for

Synthesis of C_3-symmetric star-shaped molecules containing α-amino acids and dipeptides via Negishi coupling as a key step

Sambasivarao Kotha and Saidulu Todeti

Beilstein J. Org. Chem. **2019,** *15,* 371–377. doi:10.3762/bjoc.15.33

Copies of ^{1}H, ^{13}C NMR and HRMS spectra of new compounds

License and Terms: This is a supporting information file under the terms of the Creative Commons Attribution License (http://creativecommons.org/licenses/by/4.0). Please note that the reuse, redistribution and reproduction in particular requires that the authors and source are credited.
The license is subject to the *Beilstein Journal of Organic Chemistry* terms and conditions: (https://www.beilstein-journals.org/bjoc)

^{1}H NMR of compound **10** in $CDCl_3$

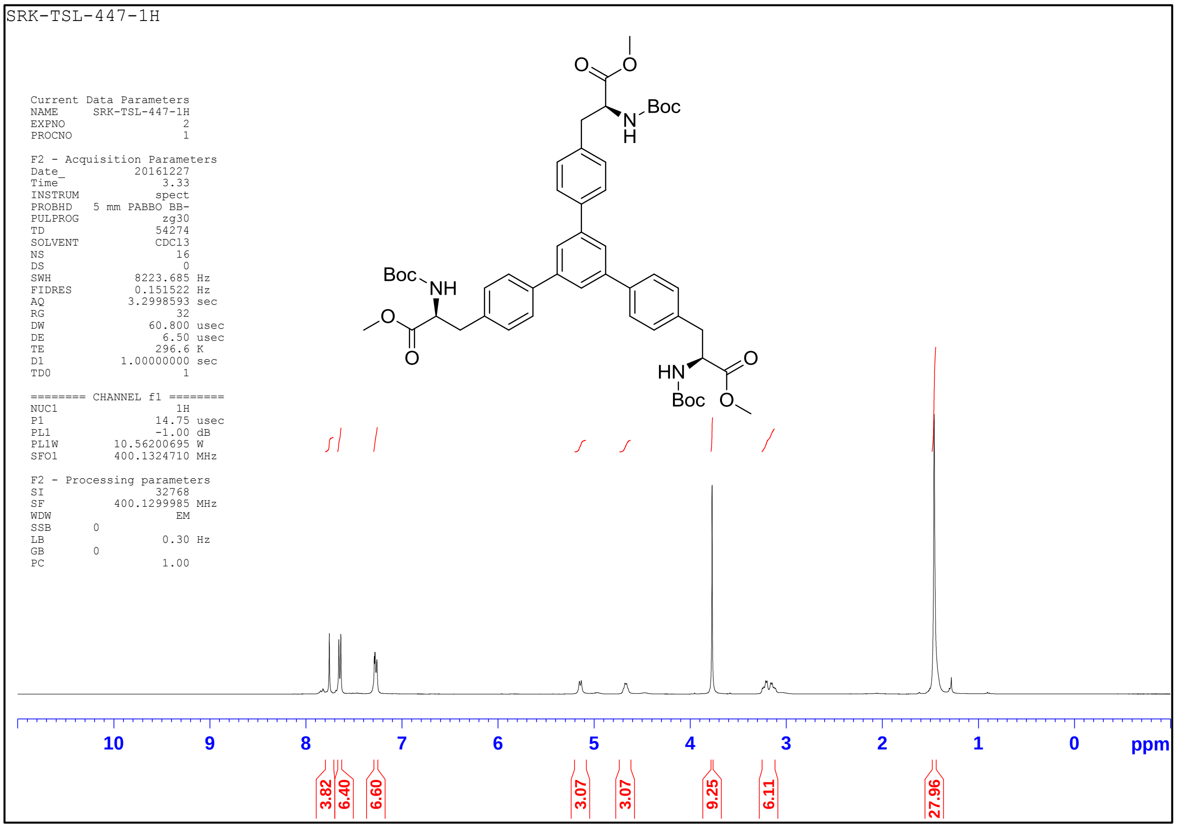

^{13}C NMR of compound **10** in $CDCl_3$

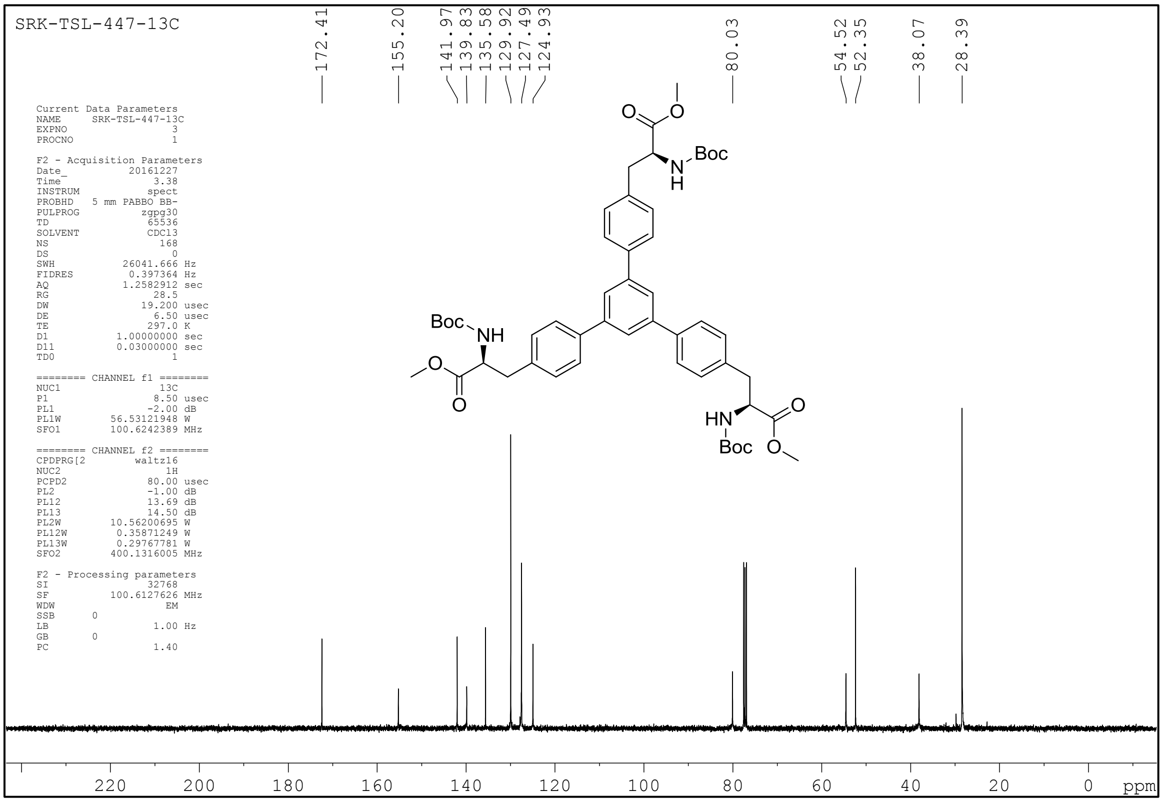

HRMS of Compound **10**

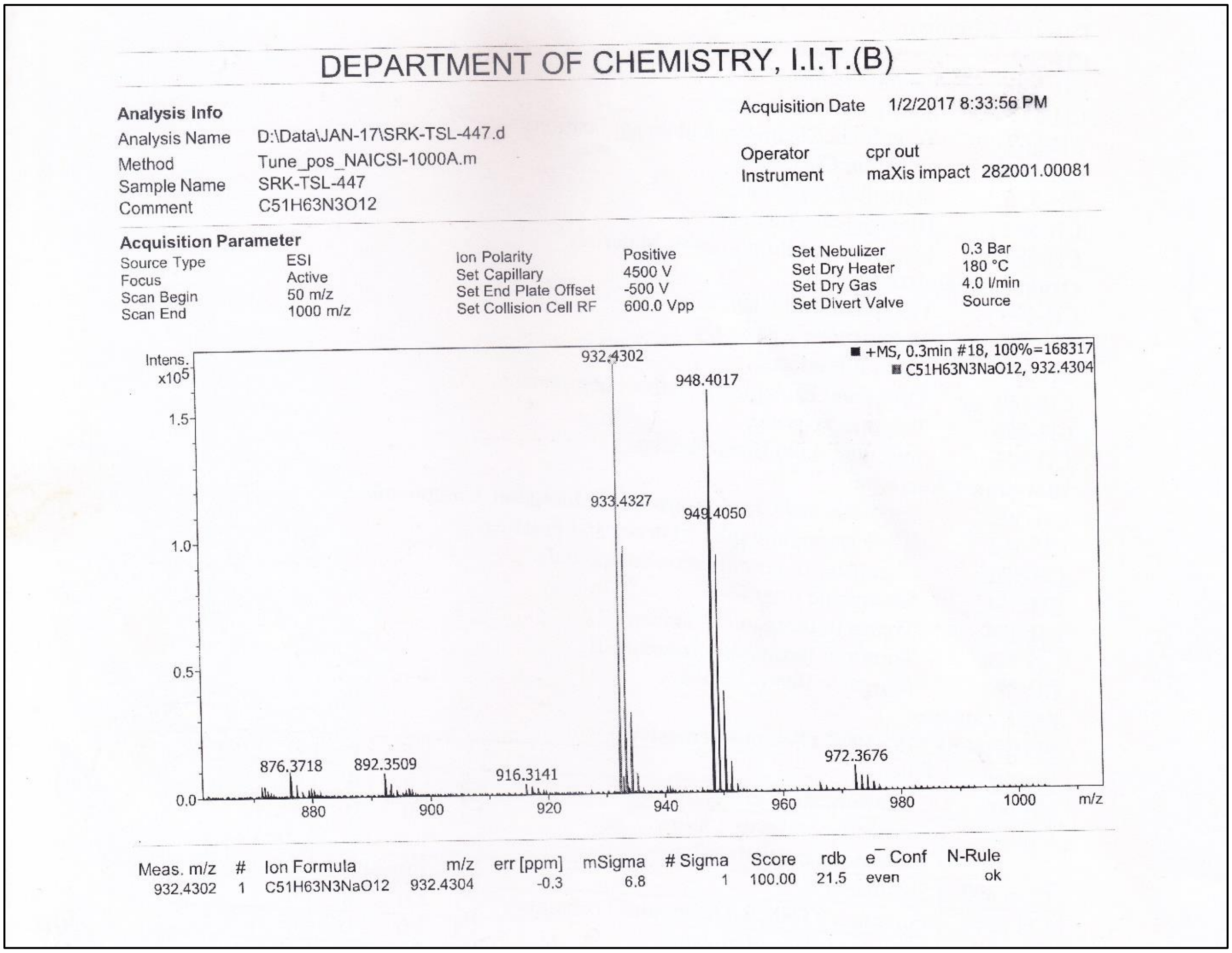

DEPARTMENT OF CHEMISTRY, I.I.T.(B)

Analysis Info

Analysis Name	D:\Data\JAN-17\SRK-TSL-447.d	Acquisition Date	1/2/2017 8:33:56 PM
Method	Tune_pos_NAICSI-1000A.m	Operator	cpr out
Sample Name	SRK-TSL-447	Instrument	maXis impact 282001.00081
Comment	C51H63N3O12		

Acquisition Parameter

Source Type	ESI	Ion Polarity	Positive	Set Nebulizer	0.3 Bar
Focus	Active	Set Capillary	4500 V	Set Dry Heater	180 °C
Scan Begin	50 m/z	Set End Plate Offset	-500 V	Set Dry Gas	4.0 l/min
Scan End	1000 m/z	Set Collision Cell RF	600.0 Vpp	Set Divert Valve	Source

Meas. m/z	#	Ion Formula	m/z	err [ppm]	mSigma	# Sigma	Score	rdb	e⁻ Conf	N-Rule
932.4302	1	C51H63N3NaO12	932.4304	-0.3	6.8	1	100.00	21.5	even	ok

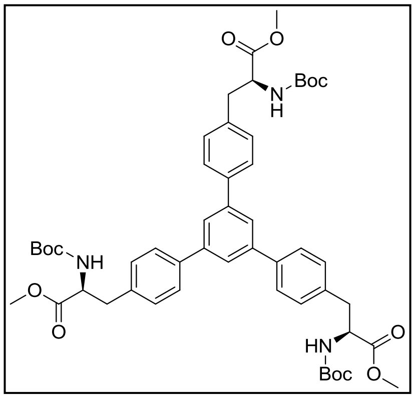

^{1}H NMR of compound **11** in $CDCl_3$

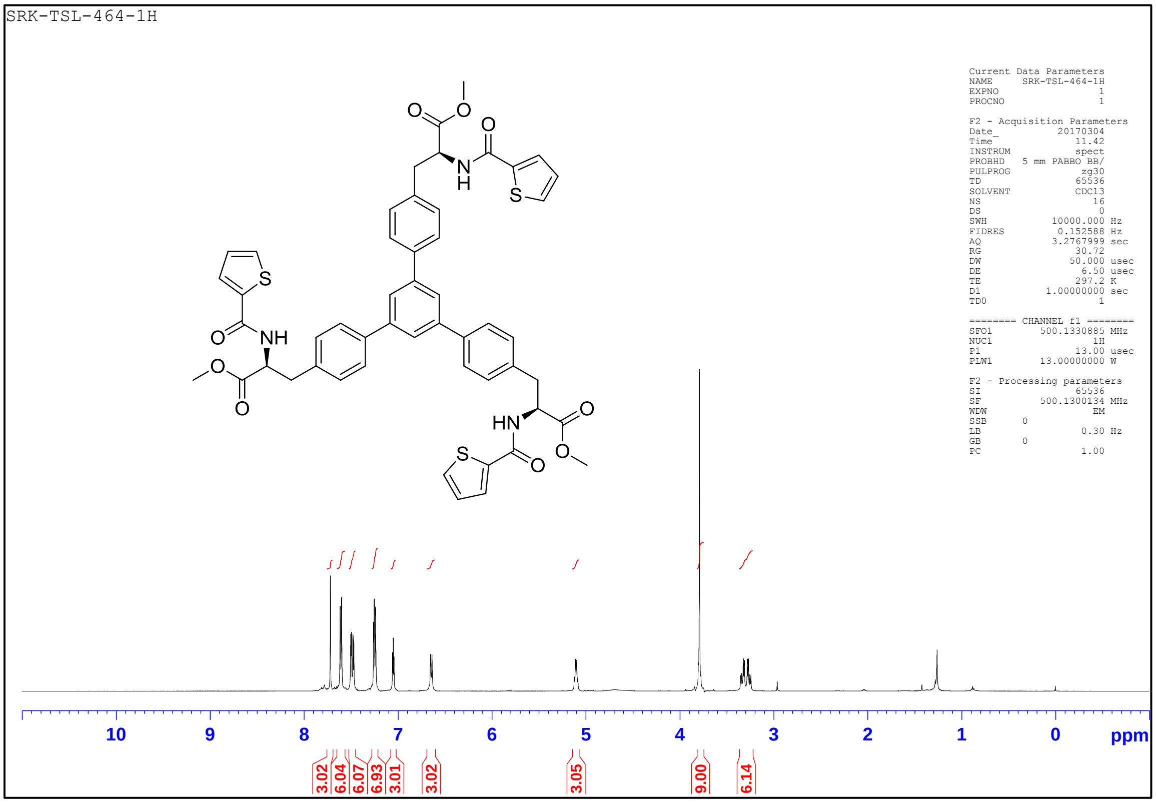

^{13}C NMR of compound **11** in $CDCl_3$

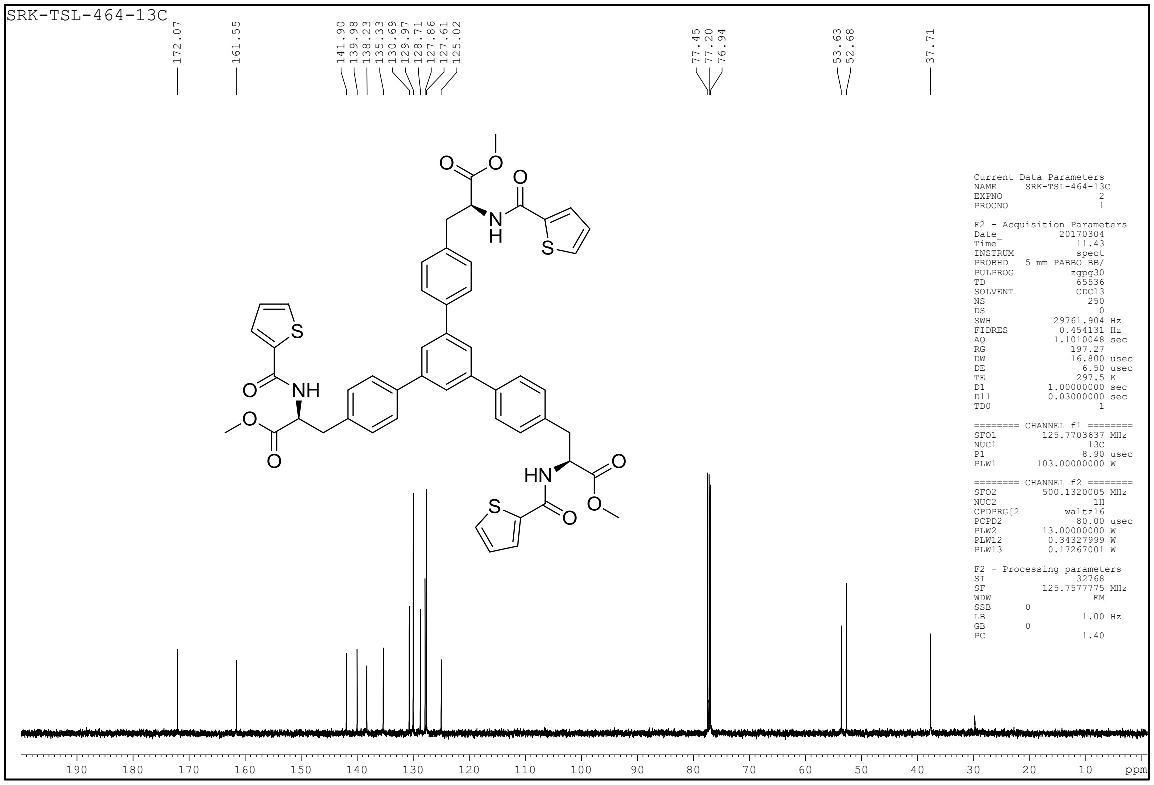

HRMS of Compound **11**

DEPARTMENT OF CHEMISTRY, I.I.T.(B)

Analysis Info

Analysis Name	D:\Data\MAR-2017\SRK-TSL-464.d	Acquisition Date	3/20/2017 12:29:39 PM
Method	Tune_pos_NAICSI-1500.m	Operator	SSKOUT
Sample Name	SRK-TSL-464	Instrument	maXis impact 282001.00081
Comment	C51H45N3O9S3		

Acquisition Parameter

Source Type	ESI	Ion Polarity	Positive	Set Nebulizer	0.3 Bar
Focus	Active	Set Capillary	4500 V	Set Dry Heater	180 °C
Scan Begin	50 m/z	Set End Plate Offset	-500 V	Set Dry Gas	4.0 l/min
Scan End	1500 m/z	Set Collision Cell RF	1800.0 Vpp	Set Divert Valve	Source

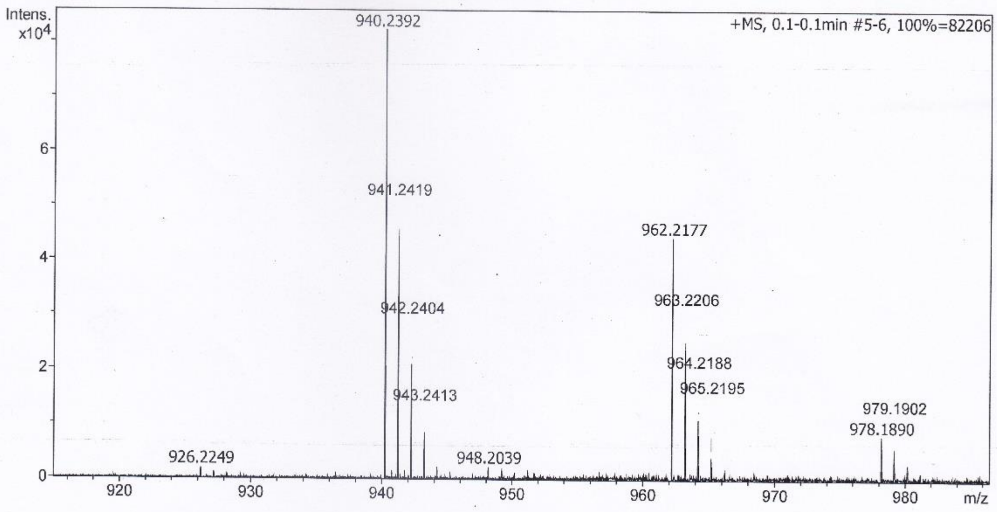

Meas. m/z	#	Ion Formula	m/z	err [ppm]	mSigma	# Sigma	Score	rdb	e⁻ Conf	N-Rule
940.2392	1	C51H46N3O9S3	940.2391	-0.1	40.7	1	100.00	30.5	even	ok

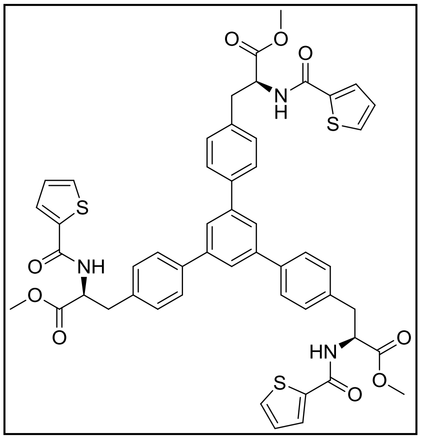

^{1}H NMR of compound **13** in $CDCl_3$

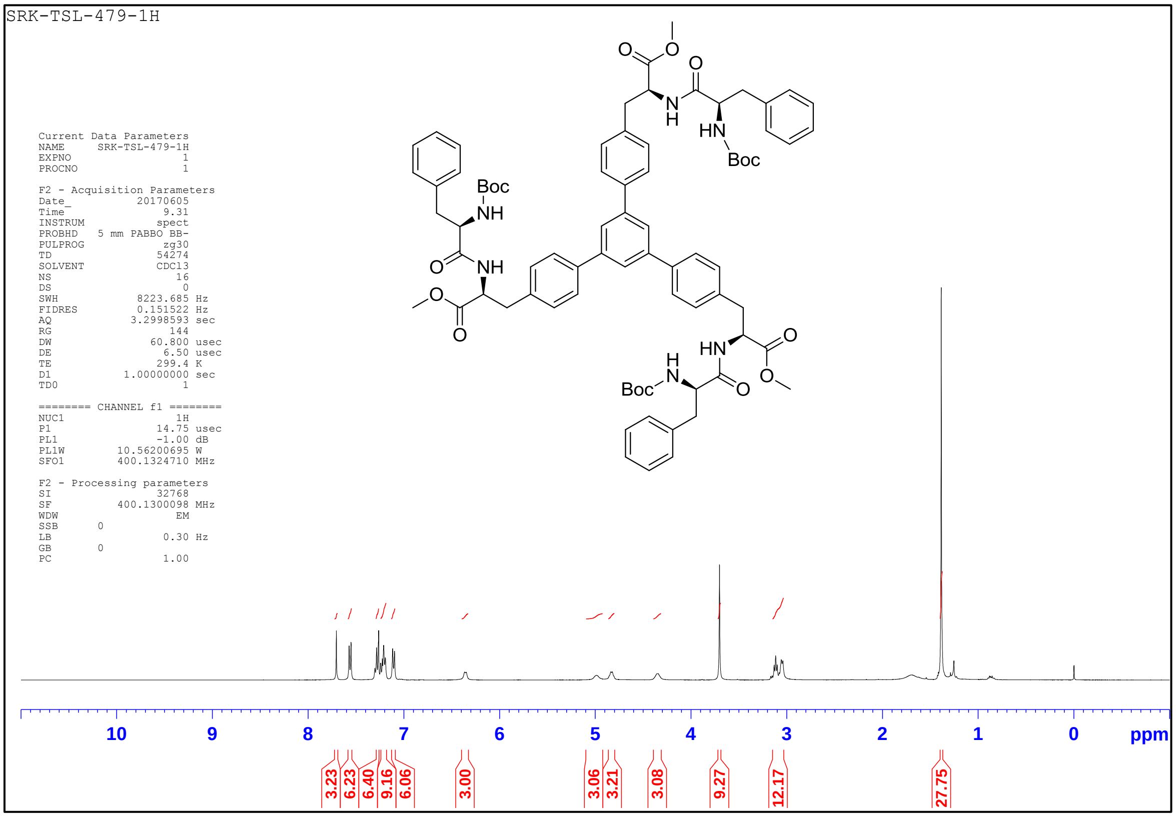

^{13}C NMR of compound **13** in $CDCl_3$

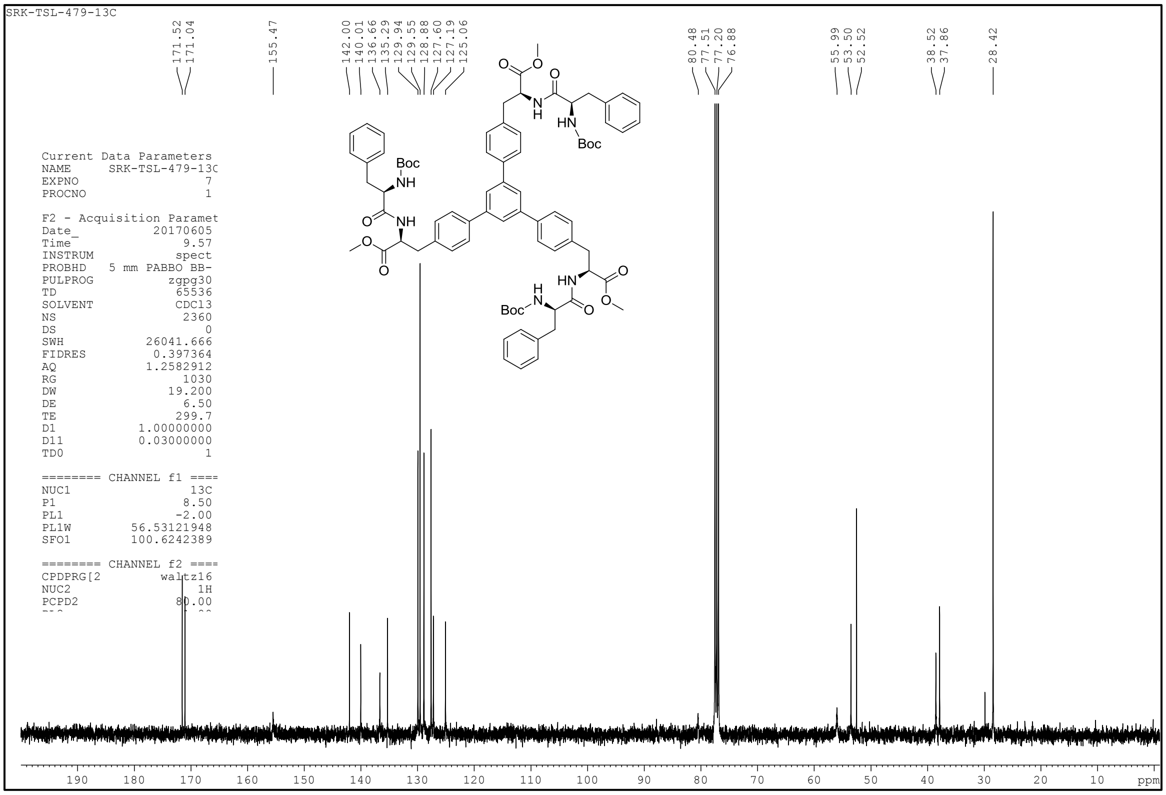

HRMS of Compound **13**

DEPARTMENT OF CHEMISTRY, I.I.T.(B)

Analysis Info

Analysis Name	D:\Data\MAY-2017\SRK-TSL-479.d	Acquisition Date	5/2/2017 6:14:47 PM
Method	Tune_pos_NAICSI-1500.m	Operator	SRK-IN
Sample Name	SRK-TSL-479	Instrument	maXis impact 282001.00081
Comment	C78H90N6O15		

Acquisition Parameter

Source Type	ESI	Ion Polarity	Positive	Set Nebulizer	0.3 Bar
Focus	Active	Set Capillary	4500 V	Set Dry Heater	180 °C
Scan Begin	50 m/z	Set End Plate Offset	-500 V	Set Dry Gas	4.0 l/min
Scan End	1500 m/z	Set Collision Cell RF	1800.0 Vpp	Set Divert Valve	Source

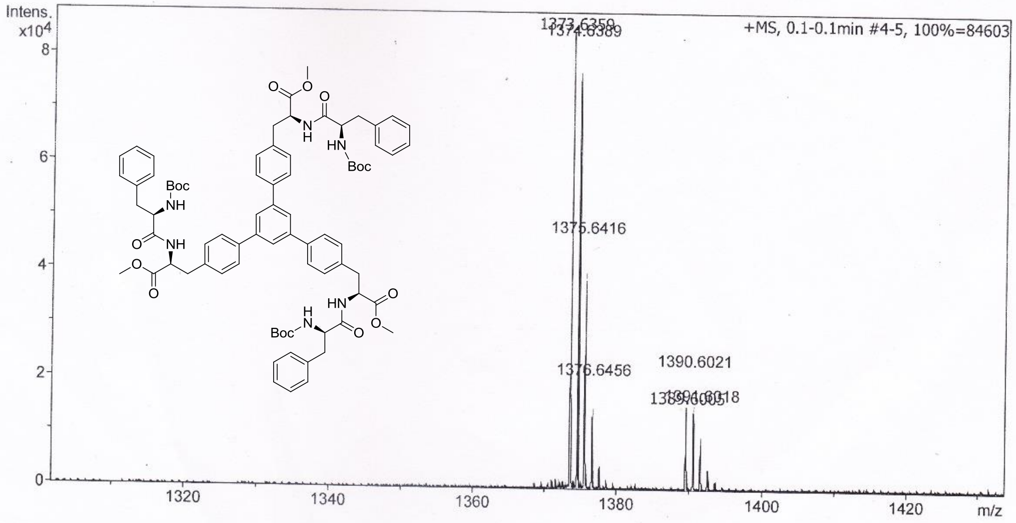

Meas. m/z	#	Ion Formula	m/z	err [ppm]	mSigma	# Sigma	Score	rdb	e⁻ Conf	N-Rule
1373.6359	1	C78H90N6NaO15	1373.6356	0.2	20.2	1	100.00	36.5	even	ok

^{1}H NMR of compound **12** in $CDCl_3$

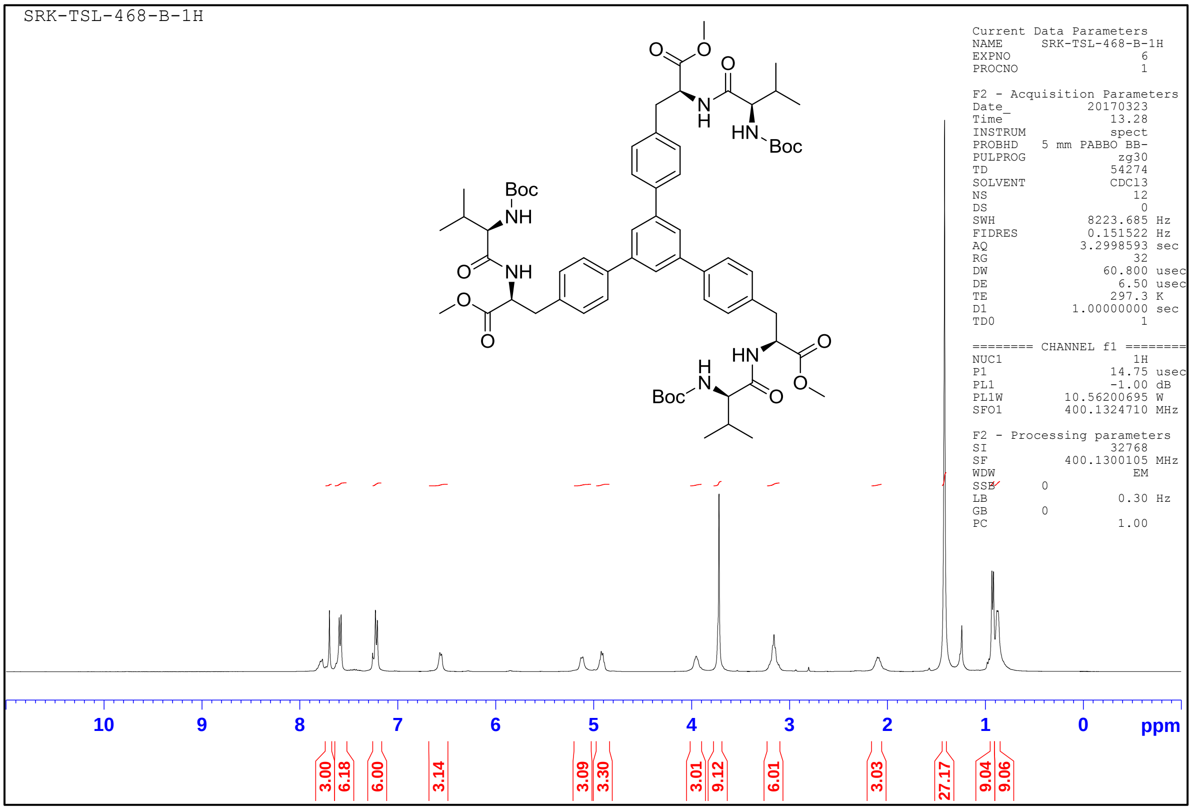

^{13}C NMR of compound **12** in $CDCl_3$

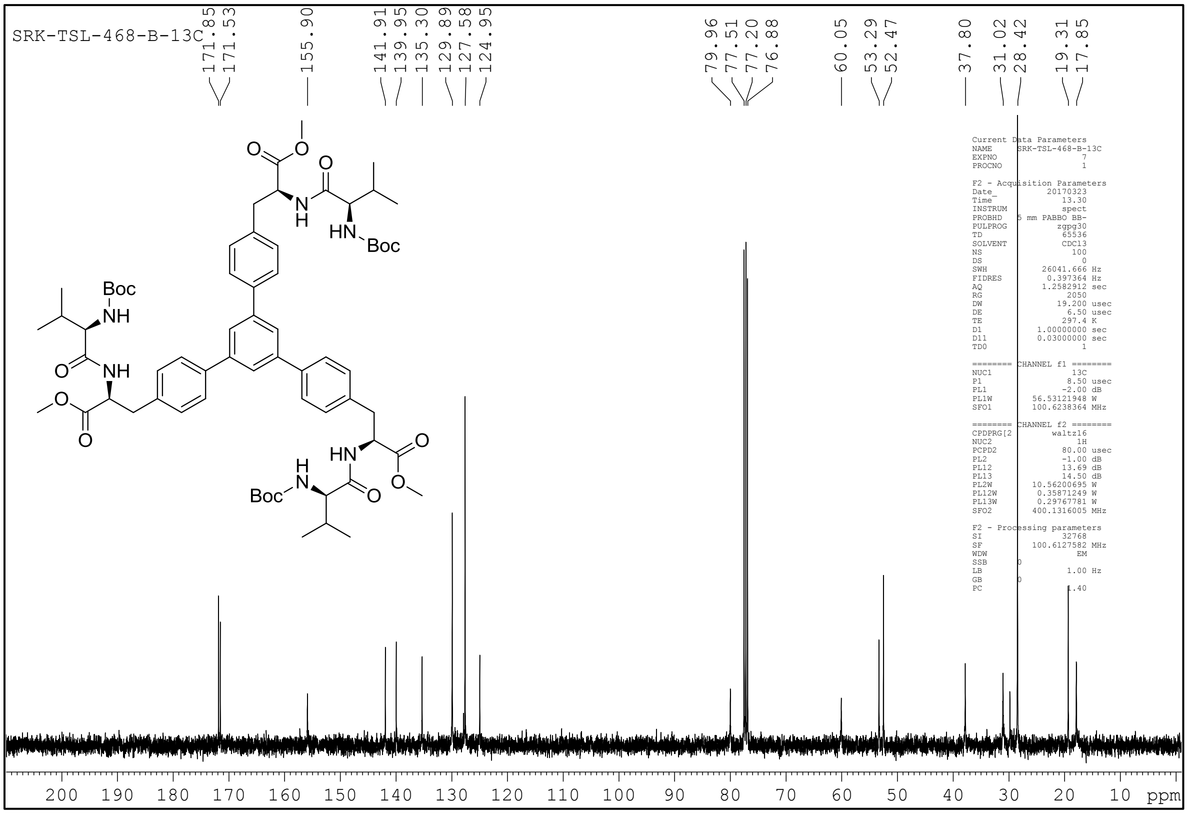

HRMS of Compound **12**

DEPARTMENT OF CHEMISTRY, I.I.T.(B)

Analysis Info

Analysis Name	D:\Data\MAR-2017\SRK-TSL-468.d	Acquisition Date	3/23/2017 9:01:06 AM
Method	Tune_pos_NAICSI-1500A.m	Operator	srk-in
Sample Name	SRK-TSL-468	Instrument	maXis impact 282001.00081
Comment	C66H90N6O15		

Acquisition Parameter

Source Type	ESI	Ion Polarity	Positive	Set Nebulizer	0.3 Bar
Focus	Active	Set Capillary	3700 V	Set Dry Heater	180 °C
Scan Begin	50 m/z	Set End Plate Offset	-500 V	Set Dry Gas	4.0 l/min
Scan End	1500 m/z	Set Collision Cell RF	1800.0 Vpp	Set Divert Valve	Source

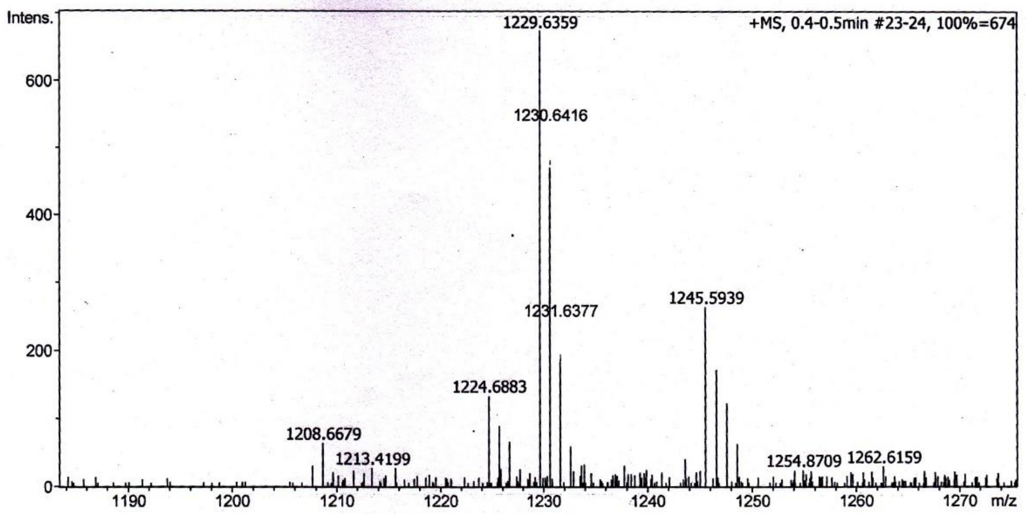

Meas. m/z	#	Ion Formula	m/z	err [ppm]	mSigma	# Sigma	Score	rdb	e⁻ Conf	N-Rule
1229.6359	1	C66H90N6NaO15	1229.6356	-0.3	28.2	1	100.00	24.5	even	ok

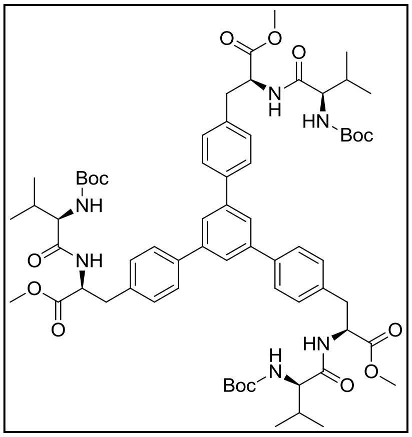

^{1}H NMR of compound **14** in MeOD

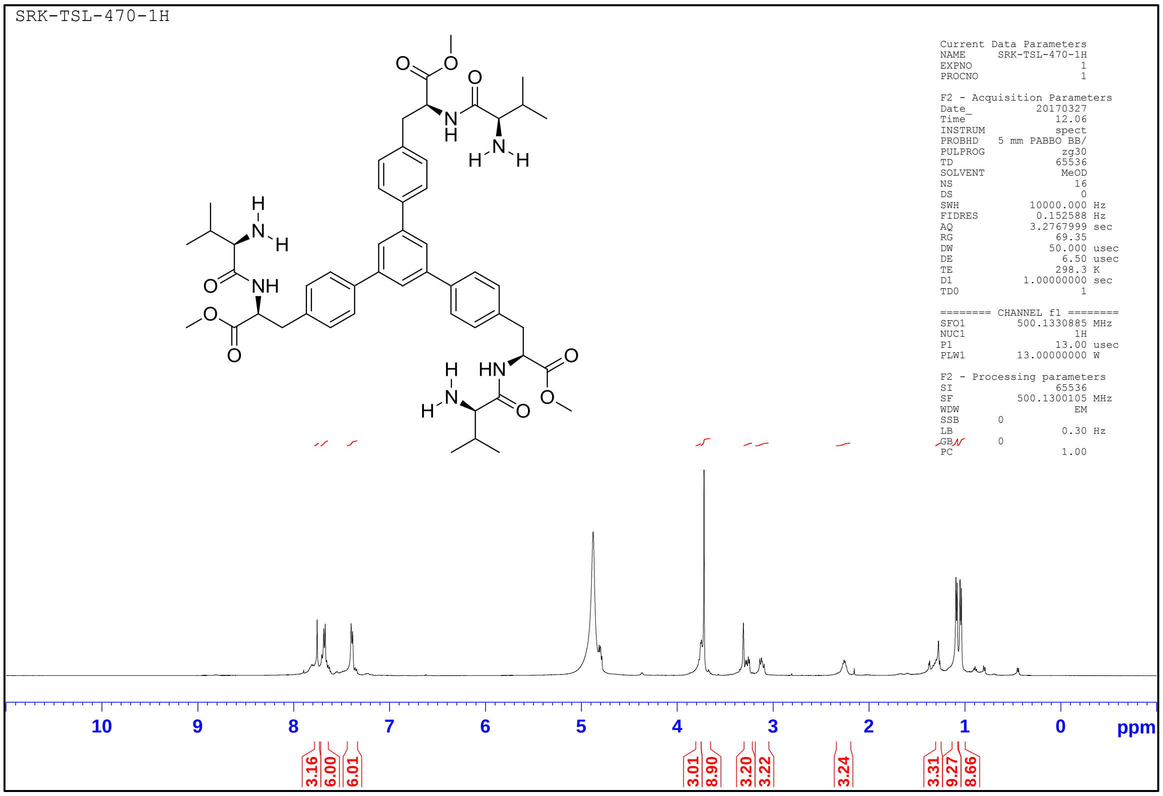

^{1}H NMR of compound **14** in MeOD

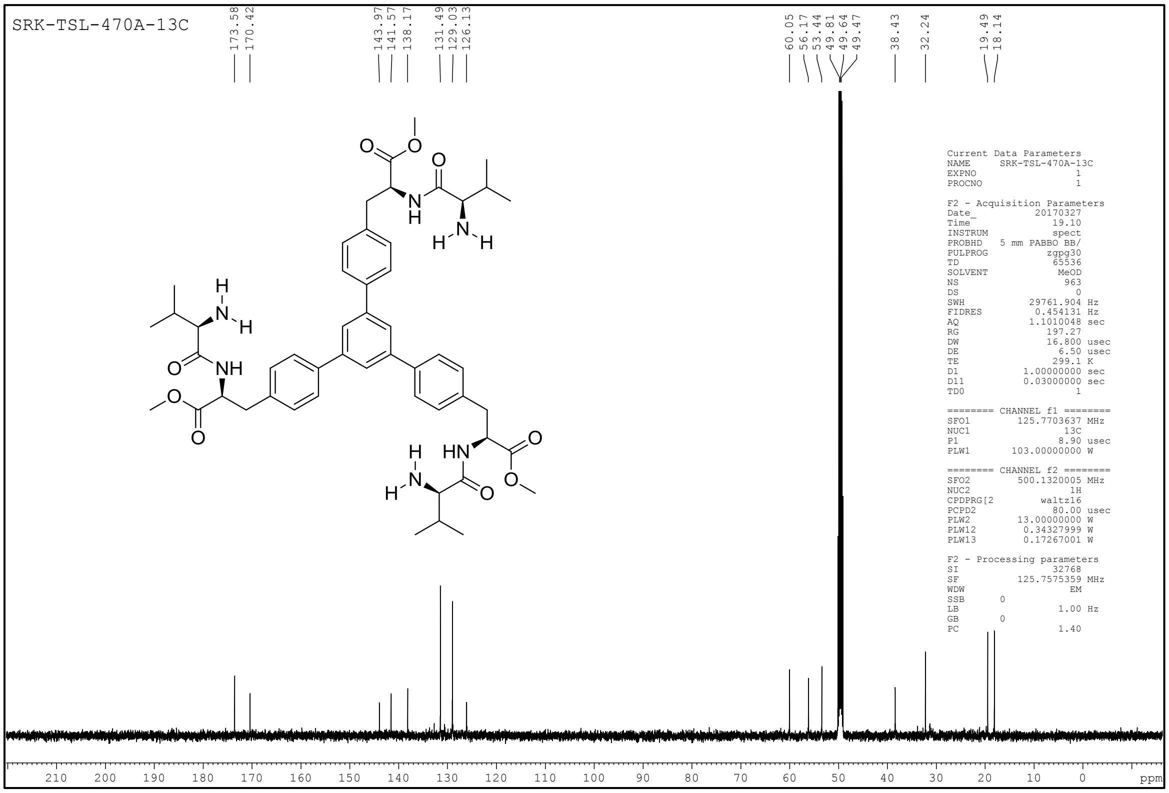

HRMS of Compound **14**

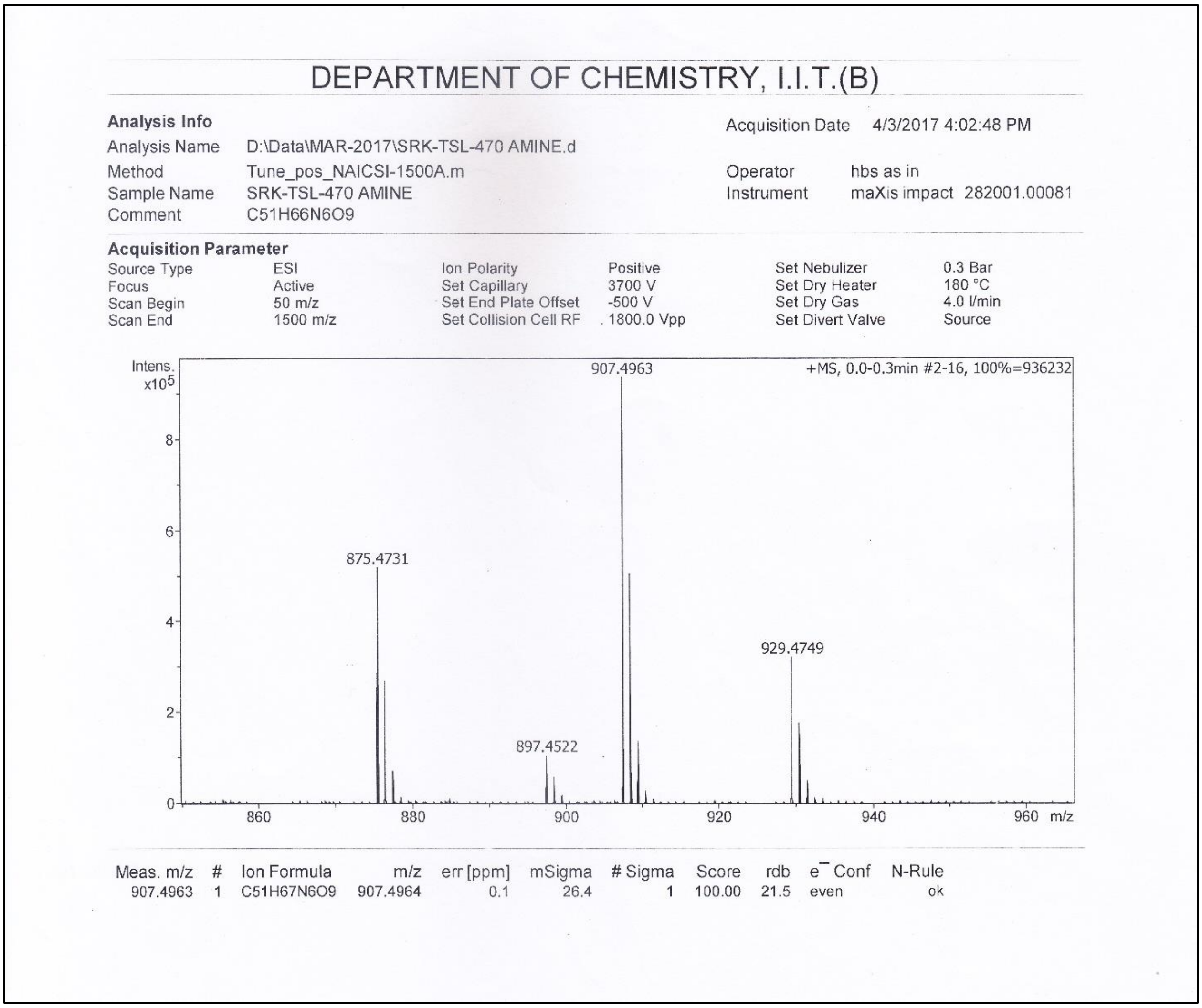

DEPARTMENT OF CHEMISTRY, I.I.T.(B)

Analysis Info

Analysis Name	D:\Data\MAR-2017\SRK-TSL-470 AMINE.d	Acquisition Date	4/3/2017 4:02:48 PM
Method	Tune_pos_NAICSI-1500A.m	Operator	hbs as in
Sample Name	SRK-TSL-470 AMINE	Instrument	maXis impact 282001.00081
Comment	C51H66N6O9		

Acquisition Parameter

Source Type	ESI	Ion Polarity	Positive	Set Nebulizer	0.3 Bar
Focus	Active	Set Capillary	3700 V	Set Dry Heater	180 °C
Scan Begin	50 m/z	Set End Plate Offset	-500 V	Set Dry Gas	4.0 l/min
Scan End	1500 m/z	Set Collision Cell RF	1800.0 Vpp	Set Divert Valve	Source

Meas. m/z	#	Ion Formula	m/z	err [ppm]	mSigma	# Sigma	Score	rdb	e⁻ Conf	N-Rule
907.4963	1	C51H67N6O9	907.4964	0.1	26.4	1	100.00	21.5	even	ok

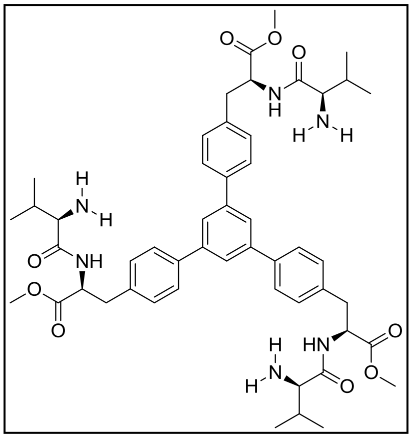

Supplement: File 1 — Copies of 1H, 13C NMR and HRMS spectra of new compounds. [file Beilstein_J_Org_Chem-15-371-s001.pdf]
